# Proton Conduction in a Single Crystal of a Phosphonato‐Sulfonate‐Based Coordination Polymer: Mechanistic Insight

**DOI:** 10.1002/cphc.202000102

**Published:** 2020-02-24

**Authors:** Ali Javed, Thorsten Wagner, Stephan Wöhlbrandt, Norbert Stock, Michael Tiemann

**Affiliations:** ^1^ Department of Chemistry Paderborn University Warburger Str. 100 33098 Paderborn Germany; ^2^ Institute of Inorganic Chemistry University of Kiel 24098 Kiel Germany

**Keywords:** coordination polymer, fuel cell, impedance spectroscopy, proton conduction, single crystal

## Abstract

The proton conduction properties of a phosphonato‐sulfonate‐based coordination polymer are studied by impedance spectroscopy using a single crystal specimen. Two distinct conduction mechanisms are identified. Water‐mediated conductance along the crystal surface occurs by mass transport, as evidenced by a high activation energy (0.54 eV). In addition, intrinsic conduction by proton ′hopping′ through the interior of the crystal with a low activation energy (0.31 eV) is observed. This latter conduction is anisotropic with respect to the crystal structure and seems to occur through a channel along the *c* axis of the orthorhombic crystal. Proton conduction is assumed to be mediated by sulfonate groups and non‐coordinating water molecules that are part of the crystal structure.

## Graphical abstract

**Anisotropic proton conduction** is observed in single crystals of a phosphonato‐sulfonate‐based coordination polymer. Inherent conductivity occurs by proton ′hopping′ through defined paths in the crystal structure. In addition, water‐mediated proton conduction at the crystal surface is identified as an additional effect.

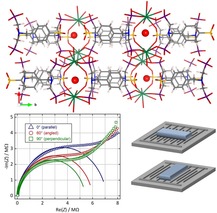

## 1 Introduction

Ion‐conducting materials have become one of the major topics in the science and technology of functional materials. They play a key role in fuel cells, which have become an integral part in modern and sustainable concepts of energy storage and conversion. For example, hydrogen (H_2_) fuel cells of the PEMFC type (proton‐exchange membrane fuel cells) require a proton‐conducting membrane as the electrolyte between anode and cathode. Perfluorosulfonic acid (PFSA) ionomers, such as Nafion^TM^ (DuPont), are most commonly used for this purpose.^1^—^3^ However, PFSA membranes exhibit some drawbacks. In addition to being rather expensive,^4^ they require a delicate humidity control for reliable and efficient operation. They show high proton conductivity only in a hydrated state (up to 21 water molecules per sulfonic acid group^5^), which is achieved by humidification of the incoming gas streams. This entails a parasitic power loss, dilution of the gases, and the risk of ′flooding′ in the system.^6^ Also, operation temperatures are restricted to *ca*. 100 °C, while higher temperatures (up to 150 °C) would be beneficial for improved efficiency and less catalyst poisoning by carbon monoxide.^6^

For the reasons stated above, there is a quest for alternative proton‐conducting materials with robust mechanical and thermal stability and reduced humidity dependency. For this matter, metal‐organic frameworks (MOFs) and other coordination polymers (CPs) have been identified as potential candidates.^7^—^11^ However, humidity still turns out to be crucial for proton conduction in most materials. This raises some questions concerning the respective proton conduction mechanism in a given material. It needs to be elucidated whether water‐mediated proton conduction really occurs through the crystal lattice, such as through channels that accommodate water molecules. Another, less favorable, possibility is that the conduction occurs predominantly along the outer surface of the grains, in which case the specific intrinsic properties of the material (porosity, taylored organic linkers, etc.) are more or less wasted. For polycrystalline materials (powders) the distinction between inherent proton conduction and surface conduction is difficult. However, when large enough single crystals are available, a single one of them can be used for proton conduction studies,^12^, ^13^, ^14^, ^15^, ^16^, ^17^ which minimizes the contribution of surface conduction and eliminates grain‐grain boundary effects. This allows to obtain valuable information concerning proton conduction paths.

Here we present impedance studies of proton conductivity in a coordination polymer single crystal. The material consists of Ba^2+^ ions connected by organic phosphonato‐sulfonate linker molecules and contains three acidic protons and two water molecules per formula unit.^18^ It exhibits a moderate proton conductivity that is humidity‐dependent. Its single‐crystallinity makes it a suitable object of study for our purpose. The results indicate that proton conduction occurs both though the crystal lattice and along the grain surface.

## 2 Results and Discussion

**Figure 1:**
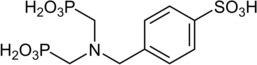
The linker molecule 4‐{[bis(phosphonomethyl)amino] methyl}benzene‐sulfonic acid ((H_2_O_3_PCH_2_)_2_N−CH_2_−C_6_H_4_−SO_3_H, H_5_L).

**Figure 2:**
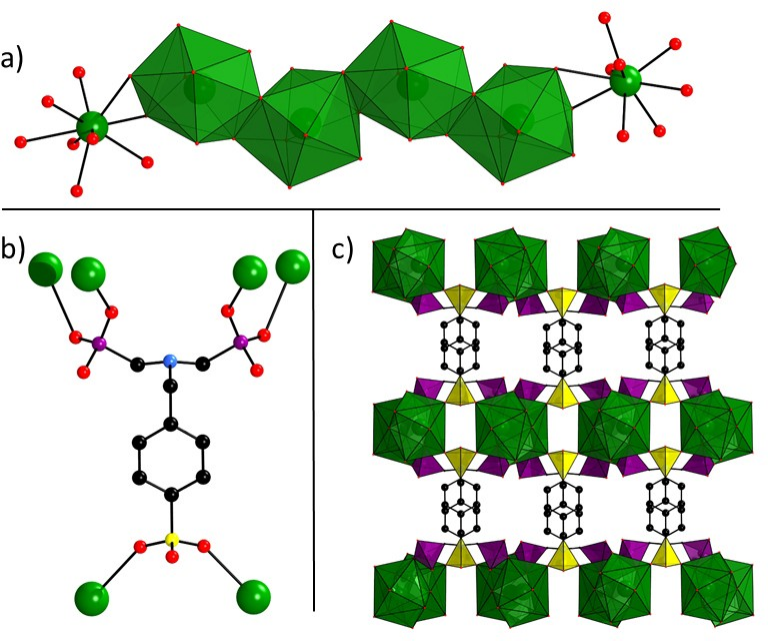
Detailed structural view at [Ba(H_3_L)(H_2_O)] ⋅ H_2_O. a) BaO_9_ polyhedra and chain along [001], b) coordination mode of the linker molecule, and c) network of [Ba(H_3_SPP)(H_2_O)] ⋅ H_2_O, view along [001] (Ba green, P magenta, S yellow, O red, C black). Hydrogen atoms omitted for clarity. Adapted from Ref. [18].

The coordination polymer [Ba(H_3_L)(H_2_O)] ⋅ H_2_O was synthesized from barium chloride (BaCl_2_⋅2H_2_O) and (4‐{[bis(phosphonomethyl) amino]methyl}benzene‐sulfonic acid, H_5_L) under solvothermal reaction conditions employing a water/ethanol mixture as the solvent.^18^ The linker molecule H_5_L (Figure 1) was prepared by sulfonation and subsequent phosphonomethylation of benzylamine, as indicated in the Experimental section. Details about the syntheses are provided in reference [18]. The coordination polymer crystallizes in the orthorhombic space group *Ama*2, as shown in Figure 2. The asymmetric unit contains one barium ion, two oxygen atoms assigned to water, and two linker molecules at a special position. Ba is surrounded by nine oxygen atoms, resulting in a mono‐capped square antiprism. The BaO_9_‐polyhedra are connected *via* edge‐sharing into a chain along [001]. One linker molecule connects a total of six Ba ions by both (PO_3_H^−^)‐groups as well as the (SO_3_^−^)‐group, thus forming a microporous, three‐dimensional network. Due to charge balance considerations, the linker is twofold deprotonated. The three remaining acidic protons are located at the hydrogen phosphonate groups and the nitrogen atom, thus forming a zwitterion. Crystallographic and experimental details, extended structure discussion and comparison is provided in reference [18].

**Figure 3:**
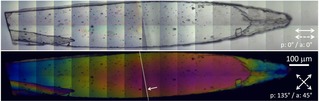
Transmission polarized light microscopic images (top: co‐polarized, bottom: cross‐polarized) of a single crystal. (Assembled from 36 single images; 36× objective lens. The small arrow indicates the edge of an underlying glass plate at the left‐hand side.

**Figure 4:**
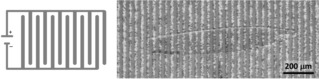
Schematic (left) of the electrode array used for contacting the single crystal and microscopic image (right) of the sample on top of the electrodes (the contours of the transparent crystal are visible).

For the investigation of the proton conductivity, we have chosen a single crystal. Polarized light microscopic images confirm the single‐crystalline nature of the specimen (Figure 3); its dimensions are 1.50 mm×0.22 mm. The thickness of the plate‐like crystal is *ca*. 14 μm, as determined by light microscopy. Characterization of the conductivity was done by impedance spectroscopy, which is the standard method for this purpose.^19^ The crystal was placed on top of an electrode array, as shown in Figure 4 and described in the Experimental Section. The contact area between electrode array and crystal was calculated based on geometric considerations (see below), assuming a smooth and planar contact area (as verified by microscopy).

This method of contacting the crystal bears a significant advantage over commonly used methods that employ gold paste or other additives for contacting:^12^, ^13^, ^14^, ^15^, ^16^ The crystal can be shifted and turned in its position which allows for stepless change of its orientation relative to the electrodes; hence, assessment of anisotropy in the proton conductivity is possible in a very straightforward way, as will be shown below.

**Figure 5:**
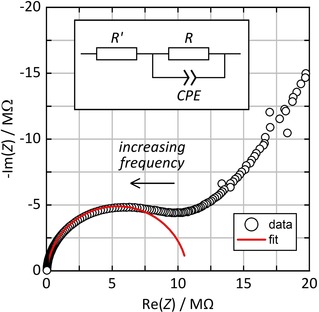
Nyquist plot of the impedance of the single crystal oriented perpendicular to the electrodes (22 °C, 90 % *r.h*.) and equivalent circuit used for fitting (*R*, *R′*: resistors, *CPE*: constant phase element).

Figure 5 shows a Nyquist plot (i. e. imaginary part *vs*. real part) of the impedance *Z* in the single crystal arranged in an orientation perpendicular to the electrodes (as shown in Figure 2) at a temperature of 22 °C and relative humidity of 90 %. The lower left part of the diagram corresponds to the high frequencies and exhibits a depressed semicircle‐like behavior. An equivalent circuit comprising two resistors (*R*, *R′*) and a constant‐phase element (*CPE*) parallel to *R* was fitted to that high‐frequency data region (between 361.22 Hz and 1 MHz). *R* then represents the proton resistance (while *R′* is attributable to extrinsic resistances, such as the contact resistance). The proton conductivity *σ* is calculated by taking into account the geometric properties of the electrode array by Equation (1), where *A* is the contact area between the sample and the electrodes, *n* is the number of spacings between electrodes, *D* is the inter‐electrode distance (20 μm), *d* is the electrode width (20 μm), and *a* is the width of the crystal:

(1) σ=n·[D/(R·A)]=n·[D/(R·a·d)]

The proton conductivity is *σ*=1.15 ⋅ 10^−4^ S cm^−1^ (at 22 °C and 90 % *r.h*.) for this crystal, which is a moderate value within the range typically observed in proton‐conducting MOFs (10^−3^ ⋅⋅⋅ 10^−5^ S cm^−1[8]^). Measurements with three different crystals under the same conditions (1.14 ⋅ 10^−4^ S cm^−1^, 1.40 ⋅ 10^−4^ S cm^−1^, 1.15 ⋅ 10^−4^ S cm^−1^) resulted in a mean value of 1.23 ⋅ 10^−4^ S cm^−1^ with a standard deviation of 1.21 ⋅ 10^−5^ S cm^−1^, which is approximately 10 %. This will be considered as the approximate error to all data in the following. (Since each measurement requires an equilibration time of 12 hours, multiple measurements were avoided for reasons of time limitations, unless stated otherwise.)

**Figure 6:**
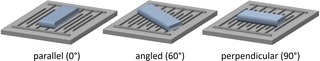
Schematic of a single crystal in variable orientation relative to the electrodes.

We have then investigated the proton conductivity of a single crystal in variable orientation relative to the electrode structure under otherwise identical conditions (Figure 6). When the long crystal axis is in perpendicular position to the electrodes, the conductivity is greater than in parallel position by one order of magnitude (Table 1). This anisotropy allows two conclusions: (i) Proton conduction is an inherent property of the crystal, *i. e*. it occurs – at least partially – inside the crystal lattice, rather than exclusively at the outer surface (such as through surface‐adsorbed water layers). In the latter case, the same conductivity would be expected for all orientations. (ii) Inherent proton conduction seems to occur preferentially in the direction along the long crystal axis. This second conclusion is further supported by the fact that the angular orientation (60°) leads to a conductivity value much closer to the perpendicular than to the parallel orientation; a conduction path along the long crystal axis can still contribute substantially in this orientation.

**Table 1:** Proton conductivities of a single crystal in variable orientation, as indicated by the schematic (22 °C, 90 % r.h.).

Orientation	Proton conductivity *σ* [S cm^−1^]
parallel (0°)	1.174 ⋅ 10^−5^
angled (60°)	8.924 ⋅ 10^−5^
perpendicular (90°)	1.142 ⋅ 10^−4^

**Figure 7:**
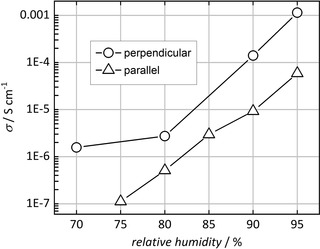
Proton conductivities of a single crystal in perpendicular and parallel orientation at variable relative humidity (22 °C).

The data presented so far were obtained at a relative humidity of 90 %. However, water turned out to have a strong impact on proton conduction. To test this impact in more detail, we have varied the relative humidity between 70 % and 95 % at a constant temperature of 22 °C, as shown in Figure 7 (logarithmic scale). The conductivity increases approximately exponentially with the relative humidity, as frequently observed in proton‐conducting coordination polymers or MOFs.^11^ Figure 5 also confirms that the conductivity is generally higher for the crystal orientation perpendicuar to the electrodes than for the parallel orientation.

Further, we have studied the impact of the temperature on the conduction properties. In theory, the proton conductivity *σ* is related to the temperature *T* by Equation (2),^9^ where *E_A_* is the activation energy, *σ*_0_ is a material‐specific factor, and *k*_B_ is the Boltzmann constant:

(2) $$\sigma =\left[\sigma {}_{0}/\left(k{}_{B}T\right)\right]\;\cdot \;\exp\left[-E{}_{A}/\left(k{}_{B}T\right)\right]$$

**Figure 8:**
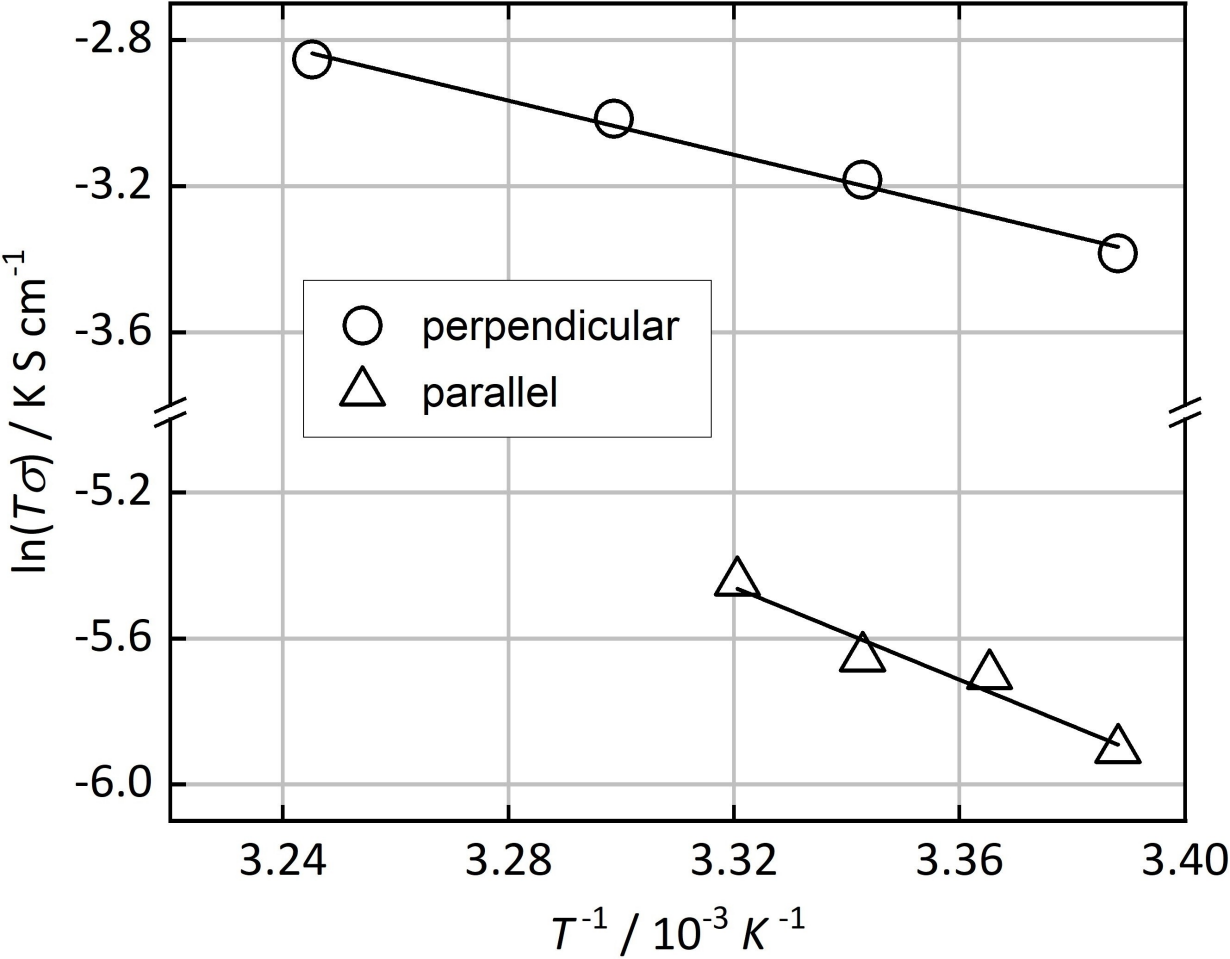
Arrhenius plots of the temperature‐dependent proton conductivities of a single crystal in perpendicular and parallel orientation (*r.h*.=90 %). The slopes of the linear fits allow to calculate the activation energies for the proton conduction (perpendicular: 0.31 eV; parallel: 0.54 eV).

Figure 8 shows the Arrhenius plots (ln(*T* ⋅ *σ*) *vs. T* ^−1^) for a single crystal in both perpendicular and parallel orientation (at a constant relative humidity of 90 %). Linear fits allow to calculate the activation energies (from the slopes, −*E_A_* ⋅ *k*_B_), which are clearly different for the two orientations. In the perpendicular orientation a fairly low activation energy of *E_A_*=0.31 eV is found, while for the parallel orientation the value is *E*_A_=0.54 eV. A low value (<0.4 eV) suggests that the proton conduction occurs by a proton ′hopping′ mechanism, such as between sulfonate groups and/or water molecules (similar to the Grotthus mechanism in bulk water).^9^ This is what we observe for the crystal orientation perpendicular to the electrodes. A high value (>0.4 eV), on the other hand, is typical of a mass transport‐based conduction mechanism, *i. e*. by diffusion of ions, such as H_3_O^+^.^20^ This seems to apply to the crystal orientation parallel to the electrodes.

The experimental findings can be summarized as follows: The [Ba(H_3_L)(H_2_O)] ⋅ H_2_O coordination polymer exhibits an altogether moderate proton conductivity. This conductivity is anisotropic with respect to the crystal axes. Along the long crystal axis (in perpendicular orientation to the electrodes) the conductivity is generally higher and marked by a low activation energy (0.31 eV) that indicates a conduction mechanism by proton ′hopping′. In the direction perpendicular to the long axis (parallel to the electrodes), the conductivity is lower and accociated with a higher activation energy (0.54 eV), which suggests a conduction mechanism marked by mass transport. In both cases an increase in humidity results in higher conductivity. To explain these findings, we propose that two distinct proton conduction phenomena occur: (i) An inherent, anisotropic conductivity through the crystal (rather than along its surfaces) exists in the direction of the long crystal axis. This conductivity occurs by proton ′hopping′. (ii) In addition, proton conduction also occurs at the crystal surfaces. This conductivity is isotropic and based on mass transport, apparently by surface‐diffusion of adsorbed water molecules; it is observed for both orientations of the crystals, which is why the conductivity is always humidity‐dependent.

**Figure 9:**
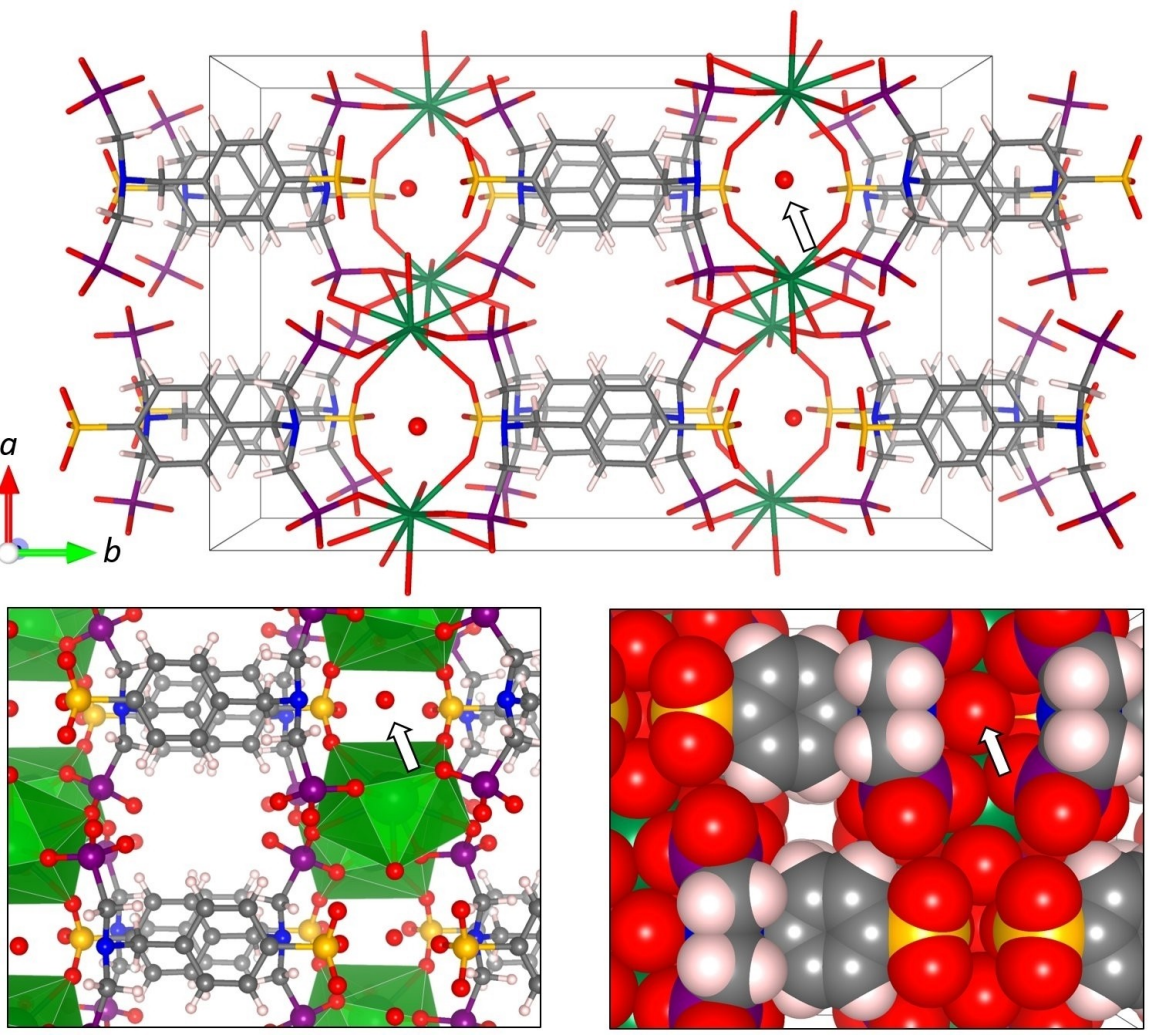
View along the *c* axis of the crystal structure (Ba green, P magenta, S yellow, O red, C grey, H white). Two types of channels in *c* direction are apparent especially in the stick (top) and ball‐and‐stick representation (bottom left). The arrow indicates the oxygen atom of the non‐coordinating water molecule. The space‐filling representation (bottom right) shows the small diameters of the channels.

To interpret the inherent (anisotropic) proton conduction mode, a closer look at the (orthorhombic) crystal structure of the coordination polymer is useful. It exhibits two distinct types of channels in the direction along the *c* axis, as shown in Figure 9. The first type of channel is flanked by the phenylene groups (with the aromatic planes in perpendicular orientation to the channel axis) and by phosphonate groups. The width of this channel is too small as to accommodate water molecules; it will therefore not contribute to water‐mediated proton conduction. The second type of channel is spanned by the Ba^2+^ cations and by sulfonate groups. It is reasonable to assume that proton conduction via proton ′hopping′ occurs through this channel, in which case the crystallographic *c* axis likely corresponds to the long axis of the elongated single crystals. (Crystal structure determination revealed that the two axes that run parallel to the large faces of the plate‐like crystals, are the *a* and *c* axes; however, they cannot be unambiguously distinguished here.) The channel contains a non‐coordinating water molecule that is quite strongly localized, which is evidenced by its small anisotropic displacement parameters. This localization is caused by H bonds to the adjacent amino group N (N ⋅⋅⋅ O distance: 2.74 Å) and phosphonate groups (O ⋅⋅⋅ OP: 2.95 Å and 2.97 Å). This water molecule and the sulfonate groups may contribute to proton ′hopping′ along the channel.

## 3 Conclusions

In summary, we have identified two distinct proton conduction mechanisms in the [Ba(H_3_L)(H_2_O)] ⋅ H_2_O (H_5_L=H_2_O_3_PCH_2_)_2_N−CH_2_−C_6_H_4_−SO_3_H) coordination polymer by measuring the impedance at variable temperature, relative humidity and crystal orientation. One proton conduction mode occurs at the crystal surface and is apparently dominated by mass transport in water adsorbate layers. The other mode is governed by a proton ′hopping′ mechanism that takes place though the interior of the crystal; it is anisotropic with respect to the crystal structure and likely occurs through a sulfonate‐lined and water‐containing channel along the *c* axis of the crystal.

## Experimental Section

The linker molecule H_5_L was prepared as described in reference [18]. In short, benzylamine was para‐sulfonated by reaction with concentrated sulfuric acid. Phosphonomethylation of the amino group was achieved with phosphonic acid and formaldehyde in half‐concentrated hydrochloric acid (*c* = 5.2 mol L^‐1^). For the synthesis of the coordination polymer [Ba(H_3_L)(H_2_O)] ⋅ H_2_O, the linker (6 mg, 16 μmol) was introduced into a 250 μl autoclave as a solid. Subsequently, 100 μl EtOH, 75 μl deionized water and 25 μl of a 1.28 mol/l solution of BaCl_2_ ⋅ 2H_2_O in deionized water were added in the given order. The reactor was closed and heated within 6 h to the reaction temperature of 150 °C. After 24 h the reactor was slowly cooled down to room temperature within 12 h. The crystals were collected via filtration and dried at ambient conditions. (Elemental analysis (%) – calculated: C 19.70, H 3.49, N 2.55, S 5.85; found: C 20.07, H 3.04, N 2.44, S 5.71.^18^)

Impedance spectra were measured by using a Solartron SI 1260 frequency response analyzer with a Chelsea 1296 Dielectric Interface. Data were recorded in the frequency range 10 Hz – 1 MHz with an input voltage amplitude of 0.1 V.^17^ An alumina substrate with a 3 mm×3 mm interdigitated Pt electrode array and an electrode width and spacing of 20 μm each (UST GmbH, Germany) was used for contacting the sample. A single crystal was placed on top of the electrode array and shifted in position with the tip of a needle under a microscope. The device was placed inside a custom‐built Faraday cage to shield the sample and improve the signal‐to noise‐ratio. The whole setup was arranged in an Espec SH‐242 climate chamber for temperature control. A constant gas stream (50 mL/min^−1^) with defined humidity was achieved by using a custom‐built gas mixing equipment based on mass flow controllers. A dry N_2_ stream (50 mL/min) was humidified by flowing through a washing bottle containing deionized water. Temperature and humidity of the gas stream were verified at the outlet of the cage by a Sensirion SHT2x sensor. The system was allowed to equilibrate for 12 hours after each change in temperature and/or humidity. The temperature of the substrate was further recorded by measuring the resistance of the Pt10 heater integrated in the alumina substrate, using an Agilent 34972 A digital multimeter. Impedance data were fitted by using ZView software. Crystal structure visualization in Figure 9 was made with VESTA software.^21^

## Supporting information

As a service to our authors and readers, this journal provides supporting information supplied by the authors. Such materials are peer reviewed and may be re‐organized for online delivery, but are not copy‐edited or typeset. Technical support issues arising from supporting information (other than missing files) should be addressed to the authors.

Supplementary
