# Supplementary material for: Proton Conduction in a Single Crystal of a Phosphonato‐Sulfonate‐Based Coordination Polymer: Mechanistic Insight
Source: Chemphyschem. 2020 Feb 24;21(7):605–9. doi: 10.1002/cphc.202000102 (PMC7187476; doi:10.1002/cphc.202000102)
Supplement: Supplementary file 1 — Supplementary [file CPHC-21-605-s001.pdf]

CHEMPHYSCHEM

Supporting Information

Proton Conduction in a Single Crystal of a Phosphonato-Sulfonate-Based Coordination Polymer: Mechanistic Insight

Ali Javed, Thorsten Wagner, Stephan Wöhlbrandt, Norbert Stock,* and Michael Tiemann*©
2020 The Authors. Published by Wiley-VCH Verlag GmbH & Co. KGaA. This is an open access article under the terms of the Creative Commons Attribution License, which permits use, distribution and reproduction in any medium, provided the original work is properly cited.

Supporting Information

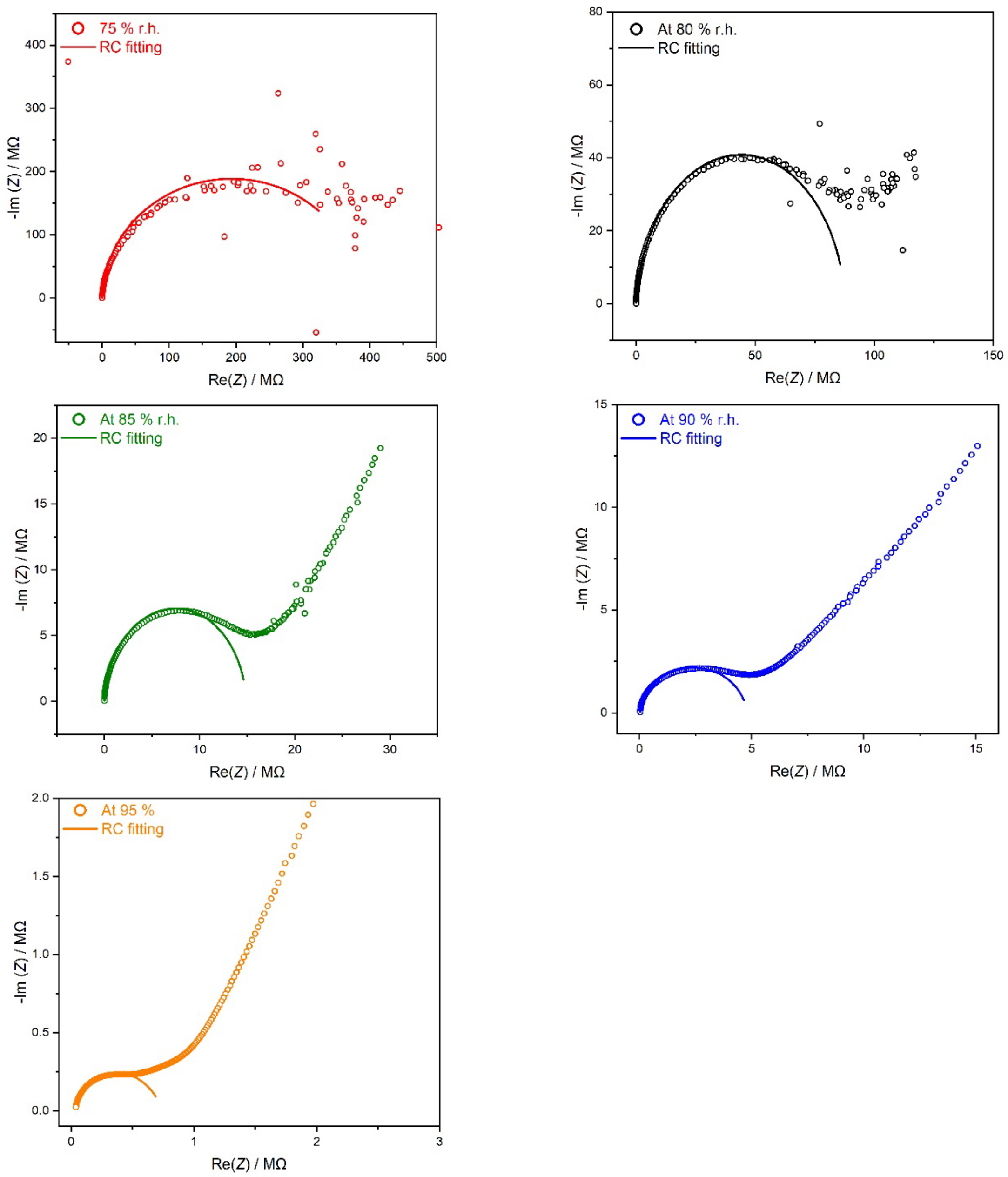

Fig. S1. Nyquist plots of single crystal oriented in <u>parallel</u> direction relative to interdigital electrodes at several setpoints of relative humidity (temperature 22 °C).

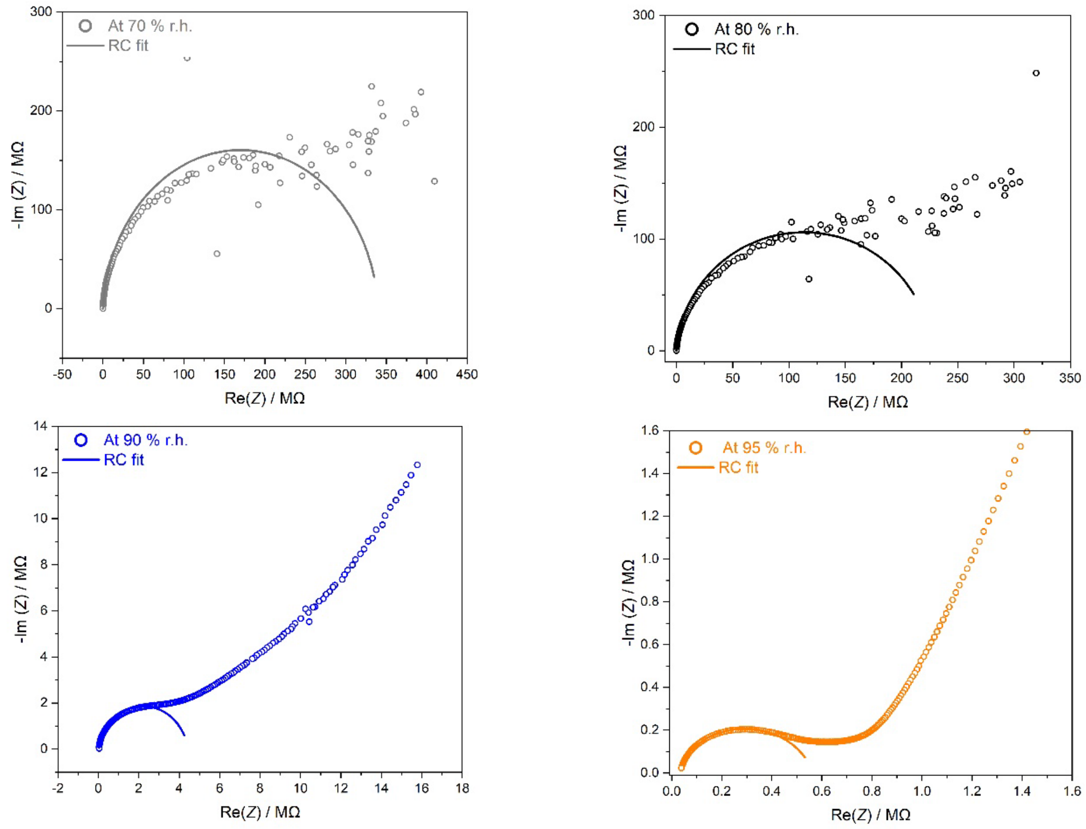

Figure S2. Nyquist plots of single crystal oriented in perpendicular direction relative to interdigital electrodes at various setpoints of relative humidity (temperature 22 °C).

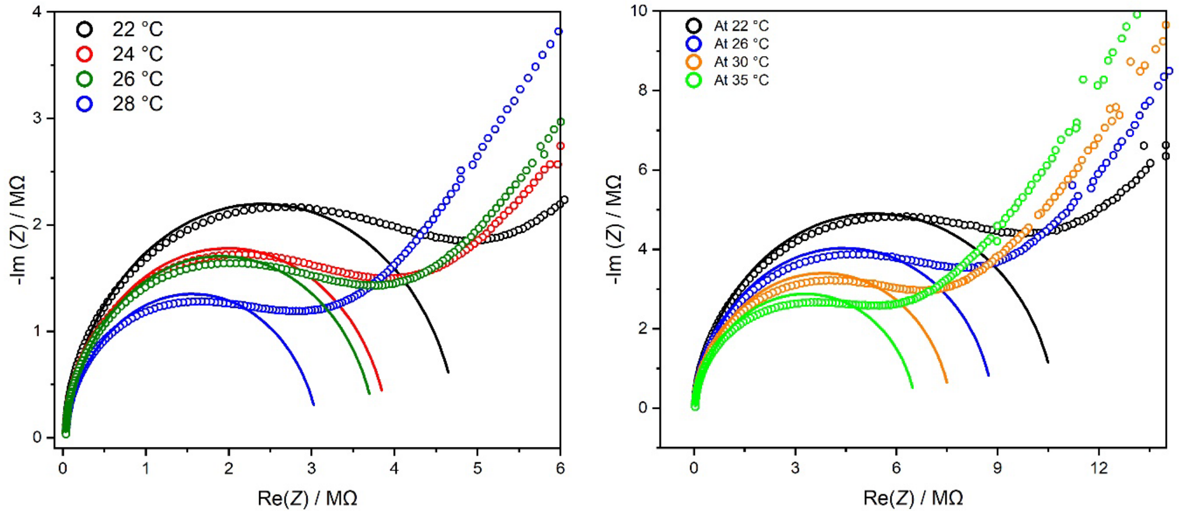

Figure S3. Nyquist plots of single crystal oriented in parallel (left) and perpendicular (right) direction relative to interdigital electrodes at variable temperature (relative humidity 90%).
